# Brain fog in postural tachycardia syndrome: An objective cerebral blood flow and neurocognitive analysis

**DOI:** 10.1002/joa3.12325

**Published:** 2020-03-03

**Authors:** Rachel Wells, Felix Paterson, Stephen Bacchi, Amanda Page, Mathias Baumert, Dennis H. Lau

**Affiliations:** ^1^ Department of Medicine Royal Adelaide Hospital Adelaide SA Australia; ^2^ Centre for Heart Rhythm Disorders University of Adelaide Adelaide SA Australia; ^3^ Centre for Nutrition and Gastrointestinal Disease University of Adelaide Adelaide SA Australia; ^4^ Department of Radiology Royal Adelaide Hospital Adelaide SA Australia; ^5^ Nutrition and Metabolism South Australian Health and Medical Research Institute (SAHMRI) Adelaide SA Australia; ^6^ School of Electrical and Electronic Engineering University of Adelaide Adelaide SA Australia; ^7^ Department of Cardiology Royal Adelaide Hospital Adelaide SA Australia

**Keywords:** cerebral blood flow velocity, cognitive dysfunction, neurovascular coupling, postural tachycardia syndrome, transcranial Doppler

## Abstract

**Background:**

It remains unclear whether brain fog is related to impaired cerebral blood flow (CBF) in postural tachycardia syndrome (POTS) patients.

**Methods:**

We assessed CBF in the posterior cerebral artery (PCA) using transcranial Doppler with visual stimuli in 11 POTS and 8 healthy subjects in the seated position, followed by neurocognitive testing.

**Results:**

CBF parameters were similar between the two groups. POTS patients demonstrated significantly longer latency in delayed match to sample response time and greater errors in attention switching task.

**Conclusions:**

Impaired short‐term memory and alertness may underlie the symptom of brain fog in POTS patients, despite normal CBF.

## 1 INTRODUCTION

Postural tachycardia syndrome (POTS) is a chronic debilitating condition in which symptoms of orthostatic intolerance are accompanied by a sustained increase in heart rate >30 bpm within 10 minutes of orthostatic challenge.^1^, ^2^ Current available treatments for POTS demonstrate only moderate efficacy with limited evidence base.^3^, ^4^, ^5^ Cognitive difficulty, often described as “brain fog”, is a prevalent complaint among patients with POTS.^6^ Interestingly, brain fog has been reported to occur even in the supine position and may not be limited to upright posture.^7^ Others have demonstrated an approximately 25% increase in cerebral blood flow (CBF) velocity in the posterior cerebral artery (PCA) of healthy individuals in response to visual stimuli.^8^ However, the CBF pattern and its response to visual stimuli have not been investigated in POTS individuals while seated. Here, we hypothesized that POTS individuals have impaired CBF regulation as well as cognition to explain the symptom of brain fog when seated.

## 2 METHODS

Consecutive patients with POTS (confirmed with 10‐minute orthostatic challenge) and complaint of brain fog (difficulty thinking/focusing/communicating, forgetful and cloudiness) were studied.^6^ All subjects remained on their usual POTS treatments. In addition, eight age‐matched healthy volunteers were studied. All patients have abstained from caffeine or alcohol for 24 hours before the study. All participants provided written informed consent. This study has institutional ethics approval.

CBF of PCA was assessed using a 2MHz phased array transducer (SonoSite Edge, SonoSite Inc). Participants were asked to sit quietly with their eyes closed for 1 minute. Pulsed Doppler mode was used to generate a real‐time image and spectral waveform of PCA CBF. Measurements of CBF velocity were taken at baseline with eyes closed for 1 minute and following a 10‐second interval after the subject was given the instruction “open your eyes”. This process was repeated five times with all recordings analyzed for percentage changes in peak systolic velocity, end diastolic velocity, and time averaged peak—a software generated measure reflecting mean CBF velocity, between eyes closed and eyes opened. Each participant was then asked to complete a series of neurocognitive tests (CANTAB: Cambridge Neuropsychological Test Automated Battery, Cambridge Cognition Ltd.) to assess executive function, reaction time, memory, and attention. These tests are described in detail elsewhere.^9^

Data were analyzed in GraphPad Prism 7. All data were not normally distributed and analyzed with Mann‐Whitney *U* test. Categorical data were compared using chi‐square analysis. A single outcome measure for latency and errors was predefined for each neurocognitive task for comparison. Statistical significance was taken as *P* < .05.

## 3 RESULTS

Nine of the 11 POTS patients were female compared with 4 of the 8 controls, with median age of 28 and 31 years, respectively (both *P* = NS). Detailed medications use is shown in the Table 1. Baseline peak systolic velocity and percentage increment of all CBF parameters with visual stimuli were similar in the PCA of POTS individuals versus healthy controls (Table 1; all *P* = NS).

**TABLE 1:** Baseline characteristics, cerebral blood flow, and neurocognitive test parameters

	POTS (n = 11)	Controls (n = 8)	***P* value**
Baseline characteristics			
Age, y (Median, IQR)	28 (19‐37)	31 (26‐35)	.3
Female, n (%)	9 (82)	4 (50)	.1
POTS medications, n (%)			
Fludrocortisone	4 (36)	—	**—**
Midodrine	5 (45)	—	
Ivabradine	3 (27)	—	
Propranolol	1 (9)	—	
Baseline peak systolic velocity, cm/s (IQR)	54 (43‐68)	49 (49‐52)	.8
Cerebral blood flow parameters: percentage increment with visual stimuli, % (Median, IQR)			
Time averaged peak	21 (15‐27)	22 (16‐24)	.9
Peak systolic velocity	15 (12‐21)	14 (9‐18)	.7
End diastolic velocity	21 (13‐24)	17 (12‐45)	.9
Neurocognitive parameters: at rest and seated (Median, IQR)			
Reaction time, ms	214 (173‐271)	227 (200‐267)	.4
Rapid visual information processing latency (ms)	437 (397‐498)	437 (410‐474)	.8
Delayed match to sample			
Latency (ms)	3630 (3029‐4395)	2482 (1754‐3295)	.04
Number of correct response (n)	4 (3‐5)	5 (4‐5)	.12
Attention switching task			
Latency (ms)	463 (425‐468)	445 (414‐487)	.6
Number of correct response (n)	156 (152‐157)	159 (158‐160)	.004

Note

IQR, interquartile range. Neurocognitive tests: The reaction time is the time taken to touch a signal as quickly as possible as it appeared in different positions on the iPad screen. The rapid visual information processing test involved identification of predefined 3‐digit sequences among rapidly appearing random numbers on the screen. The delayed match to sample task instructs the subject to identify which of 4 images provided is identical to the target image shown previously. Latency is the time taken to touch the correct image on the screen. The attention switching task instructs the subject to touch the correct response to answer a prompt word that appears above an arrow. It measures the subject's ability to ignore irrelevant information when responding to a question. Latency is the time taken to touch the correct response.

POTS individuals had a significantly longer latency in delayed match to sample response (3.6 vs 2.5 seconds, *P* = .04) and achieved lower number of correct responses during attention switching tasks as compared with healthy controls. However, the median latencies for the reaction, attention switching, and rapid visual information processing tests were not statistically significant (Table 1).

## 4 DISCUSSION

This study demonstrated objective evidence of neurocognitive deficits in POTS individuals but similar increment in CBF velocity parameters in response to visual stimuli in the PCA of both groups. Others have demonstrated a correlation between increment in CBF in the middle cerebral artery (MCA) during cognitive challenge (functional hyperemia) and the ability of POTS patients to recall numbers. The deficit in functional hyperemia became much more pronounced when the cognitive challenge was performed concurrently with orthostatic challenge, suggesting impairment of both neurovascular coupling and autoregulation.^7^ Autoregulation tends to deteriorate in patients with POTS during orthostatic challenge, as oscillations in peripheral blood pressure become more marked.^7^, ^10^ We found normal CBF velocity response in the PCA to visual stimuli in POTS patients while remaining seated. However, it remains unknown whether CBF changes would differ if measured in other vessels such as the MCA or with more complicated visual search paradigms.^11^

There has been an increasing awareness of cognitive dysfunction in POTS patients. To date, formal neurocognitive assessments in POTS patients remain limited with variable findings of deficits in memory, attention, and executive function using different neuropsychological testing tools.^12^ We found deficits in short‐term memory and alertness in our POTS cohort. In contrast, others have shown impaired selective attention and cognitive processing but unaffected memory in POTS patients using different neuropsychological tests.^13^ This may be owing to the heterogenous nature of the condition and the many factors such as sleep disturbances, chronic fatigue, and medication use that may influence different facets of the cognitive status. These objective measures of cognitive dysfunction may in part explain the brain fog described by POTS patients even when recumbent, although the mechanisms remain unclear.^6^

### 4.1 Study limitations

Headgear fixation of ultrasound probe was not adopted to reduce variability in CBF measurements and CBF was not measured during cognitive testing because of the lengthy protocols (45 minutes) and the challenge of maintaining a consistent angle of insonation to the PCA with handheld probe. Furthermore, end‐tidal carbon dioxide levels were not measured in this study. The lack of significance in some cognitive parameters may be caused by the small number of POTS subjects in this study.

## 5 CONCLUSION

POTS patients demonstrate normal CBF velocity increment in response to visual stimuli when seated. Impaired short‐term memory and alertness may reflect the symptom of brain fog in POTS patients.

## STATEMENT OF ETHICS

This work was approved by the institutional human research ethics committee and conducted ethically in accordance with the World Medical Association Declaration of Helsinki.

## CONFLICT OF INTEREST

The University of Adelaide reports having received on behalf of Dr Lau lecture and/or consulting fees from Abbott Medical, Bayer, Boehringer Ingelheim, Biotronik, BMS Pfizer, and Medtronic.

## AUTHOR CONTRIBUTIONS

Study conception or design: RW, FP, & SB; Data acquisition & analysis: RW, FP, & SB; Data interpretation: RW, AP, MB, & DHL; Drafting & critical review: all authors; Final approval of study: all authors; Accountability for all aspects of work: all authors.
